# Substance use and adolescents’ activities in digital space

**DOI:** 10.1192/j.eurpsy.2025.800

**Published:** 2025-08-26

**Authors:** L. Csémy, L. Kážmér, P. Baďura

**Affiliations:** 1 National Institute of Mental Health, Klecany; 2 Palacký University, Olomouc, Czech Republic

## Abstract

**Introduction:**

Representative surveys have shown a decline in alcohol and tobacco use among adolescents over the last years. The rapid spread of adolescents’ activities in digital space may have contributed to these changes. However, there is still considerable scope for examining the relationship between activities in digital space and substance use behaviour of adolescents.

**Objectives:**

The study aims to assess the association between adolescents’ alcohol use, cigarette smoking and their activities in digital space. The respective associations are controlled for adolescents’ subjective well-being as a putative confounder related to both substance use and digital activities.

**Methods:**

Data from the 2022 Czech part of the *Health Behaviour in School-aged Children* study were used. Only 15-year-old respondents were analysed (*N*=4263, 50.7% boys). To measure substance use, data on the frequency of alcohol use and cigarette smoking in the past 30 days were used. Digital activities were measured by data on typical daily free time spent on social media and computer gaming. To control for subjective well-being the WHO-5 scale was used. Pairwise associations between substance use, social media use, and computer gaming were examined using Spearman rank correlation coefficients (*rho*). Ordinal logistic regression models were used to adjust the respective associations for gender and psychological well-being.

**Results:**

Significant pairwise associations were found between time spent on social media and both frequency of alcohol use (*rho* = 0.181, *P*<0.05) and cigarette smoking (*rho* = 0.191, *P*<0.05). In contrast, computer gaming was not significantly correlated with either alcohol use or cigarette smoking (*rho* = -0.015 and *rho* = -0.014, respectively; *P*>0.30). Poor subjective well-being was a significant risk factor for substance use and time spent on social media. Controlling for adolescents’ well-being did not significantly attenuate the former relationship between the substance use and social media use.

**Conclusions:**

The results suggest that among adolescents, different forms of digital activity have markedly different associations with substance use. While increased time spent on social media was significantly associated with higher frequency of substance use, no such association was found for computer gaming. Future research will focus on whether the relationship between specific digital activities and substance use can be explained by other factors related to mental health.

**Funding:**

The study was supported by the OP Johannes Amos Comenius (CZ.02.01.01/00/22_008/0004583).

**Disclosure of Interest:**

None Declared

